# Circulating calprotectin and S100A8/A9-related biomarkers for diagnosis and prognostic stratification in adult acute infection and sepsis: a systematic review and meta-analysis

**DOI:** 10.3389/fcimb.2026.1934020

**Published:** 2026-10-14

**Authors:** Yin-sheng Liao, Yu-ping Deng, Jian-li Song, Xuan Yu, Qiang Li, Bin Lu, Guan-yu Chen

**Affiliations:** 1 Department of Anesthesiology, Zigong Fourth People’s Hospital, Zigong, Sichuan, China; 2 Department of Anesthesiology, Zigong Hospital of Chengdu University of Traditional Chinese Medicine, Zigong, Sichuan, China

**Keywords:** acute infection, biomarker, calprotectin, critical care, diagnostic accuracy, meta-analysis, S100A8/A9, sepsis

## Abstract

**Background:**

Circulating calprotectin is a proposed marker of acute innate immune activation, but reported diagnostic and prognostic performance is inconsistent. We assessed circulating calprotectin and S100A8/A9-related biomarkers for diagnosing acute infection and sepsis and stratifying prognosis in adults.

**Methods:**

We searched biomedical databases and trial registries through July 4, 2026, for adult studies of circulating calprotectin/S100A8/A9 in acute infection or sepsis. Random-effects models pooled diagnostic and reported prognostic areas under the receiver operating characteristic curve (AUC). Threshold-based sensitivity and specificity were analyzed separately using bivariate random-effects models. We used the Quality Assessment of Diagnostic Accuracy Studies-2 (QUADAS-2) and Grading of Recommendations Assessment, Development and Evaluation (GRADE) to assess diagnostic risk of bias and evidence certainty. Exploratory subgroup, sensitivity, biomarker-comparison, and combined-model analyses were also performed.

**Results:**

Twenty-two reports representing 21 independent cohorts were included. The primary diagnostic meta-analysis yielded a pooled AUC of 0.741 (95% confidence interval [CI], 0.657–0.810). The pooled AUC was 0.703 (95% CI, 0.600–0.789) for sepsis and 0.758 (95% CI, 0.636–0.849) for acute infection or infection-source-related outcomes. In separate threshold-based analyses, pooled sensitivity and specificity were 0.70 (95% CI, 0.46–0.86) and 0.57 (95% CI, 0.55–0.59) for sepsis, and 0.46 (95% CI, 0.09–0.88) and 0.86 (95% CI, 0.60–0.96) for acute infection or infection-source-related outcomes, respectively. For mortality/survival outcomes, pooled sensitivity and specificity were 0.72 (95% CI, 0.65–0.77) and 0.66 (95% CI, 0.53–0.77), respectively, while the pooled reported prognostic AUC was 0.737 (95% CI, 0.688–0.786). Exploratory analyses were directionally stable, but paired biomarker comparisons did not provide definitive evidence of superiority. Certainty was very low for all major outcomes.

**Conclusions:**

Circulating calprotectin and S100A8/A9-related biomarkers provide moderate, context-dependent information in adult acute infection and sepsis. They may serve as adjunctive host-response markers, particularly for selected infection/source contrasts, but current evidence does not support stand-alone diagnosis, universal thresholds, or replacement of established biomarkers and clinical assessment.

**Systematic Review Registration:**

https://www.crd.york.ac.uk/PROSPERO/view/CRD420261441569, identifier CRD420261441569.

## Background

Timely recognition of infection and sepsis remains challenging in emergency and intensive care settings. Sepsis is life-threatening organ dysfunction caused by a dysregulated host response to infection and remains a major contributor to global mortality (Singer et al., 2016; Rudd et al., 2020). Infection may present with non-specific systemic inflammation, whereas sterile inflammation after surgery, trauma, burns, intoxication, malignancy, or critical illness can closely mimic infection. Delayed antimicrobial treatment may worsen outcomes in sepsis, while unnecessary exposure increases antimicrobial-resistance and stewardship pressures (Evans et al., 2021; Antimicrobial Resistance Collaborators, 2022).

Current biomarkers do not fully resolve this diagnostic uncertainty. C-reactive protein (CRP) is widely available but rises relatively late and lacks specificity for infection. Procalcitonin (PCT) may better reflect bacterial infection, but its performance varies with infection site, renal function, immune status, postoperative inflammation, and disease severity (Wacker et al., 2013; Barichello et al., 2022). Lactate, the Sequential Organ Failure Assessment (SOFA) score, and other severity measures support risk stratification but do not directly distinguish infection from sterile inflammation (Singer et al., 2016; Barichello et al., 2022; Zimmermann et al., 2024). An early host-response biomarker that complements routine inflammatory markers could therefore improve clinical assessment. Calprotectin, a heterodimer of S100A8 and S100A9, is abundant in neutrophils and monocytes and is released during myeloid-cell activation and inflammatory tissue injury (Wang et al., 2018). Circulating calprotectin may thus reflect innate immune activation more directly than hepatic acute-phase proteins. However, myeloid activation is not infection-specific, and calprotectin may also increase during sterile inflammation, tissue injury, and immune dysregulation. Its clinical value therefore depends on its ability to distinguish clinically relevant diagnostic states rather than merely identify inflammation.

Reported diagnostic performance varies across populations and target conditions, with favorable findings in some bacterial or respiratory infections but more modest accuracy in broader emergency and intensive care cohorts (Bakalar et al., 2026; Bartakova et al., 2019; Christensen et al., 2022; Diehl-Wiesenecker et al., 2024; Jonsson et al., 2017; Larsson et al., 2020; Lengquist et al., 2025; Sejersen et al., 2025b; Wollmer et al., 2021, 2026; Zimmermann et al., 2026). This variation may reflect differences in population spectrum, reference standards, specimens, assays, analyte definitions, cut-offs, and target conditions. A previous meta-analysis included only six intensive care unit records, combined adult and neonatal populations, and searched through January 2022 (Gao et al., 2022). Several subsequent adult studies have reported heterogeneous and often less favorable findings across diverse clinical settings.

We therefore conducted an updated systematic review and meta-analysis of circulating calprotectin and S100A8/A9-related biomarkers for diagnosing and prognostically stratifying acute infection and sepsis in adults. The primary diagnostic synthesis was restricted to clinically relevant suspected-infection or suspected-sepsis comparator settings; studies using special controls, pathogen- or site-specific designs, or severity-only endpoints were evaluated separately in exploratory analyses.

## Methods

### Review design and reporting

This systematic review and meta-analysis followed the Preferred Reporting Items for Systematic Reviews and Meta-Analyses (PRISMA) and PRISMA for Diagnostic Test Accuracy Studies (PRISMA-DTA) guidance (Page et al., 2021; McInnes et al., 2018). The protocol was registered in PROSPERO (CRD420261441569). We evaluated the diagnostic and prognostic performance of circulating calprotectin and S100A8/A9-related biomarkers in adults with suspected or confirmed acute infection, sepsis, septic shock, or relevant non-infectious inflammatory mimics. The completed PRISMA-DTA checklist is provided in **Supplementary File 1**.

### Eligibility criteria

Eligibility criteria were defined using the Population, Index test, Comparator, Outcomes, and Study design (PICOS) framework:

Population: adults (≥18 years) with suspected or confirmed acute infection, bacterial infection, sepsis, septic shock, or relevant non-infectious inflammatory mimics in acute-care settings.

Index test: blood, plasma, or serum total calprotectin, S100A8/A9, S100A8, or S100A9, with baseline or early sampling prioritized for diagnostic analyses.

Comparator: clinically relevant suspected-infection, suspected-sepsis, non-infectious inflammatory, viral, non-bacterial, or low-probability infection groups for the main diagnostic analysis.

Outcomes: acute or bacterial infection, sepsis, bloodstream infection, lower respiratory tract infection, or infection-source differentiation for diagnosis; mortality/survival or severity-related endpoints for prognosis.

Study design: prospective or retrospective diagnostic accuracy, cohort, nested case-control, clinical prediction, or prognostic studies reporting extractable performance data.

We excluded pediatric-only, animal, *in-vitro*, fecal or local-fluid biomarker studies, non-original publications, and studies without extractable data. Healthy-control, special-control, single-pathogen, highly site-specific, and severity-only studies were retained for exploratory analyses when they did not meet the main clinical-comparator criteria. Detailed eligibility and evidence-set classification rules are provided in **Supplementary File 4**.

### Search strategy

Two reviewers (YL and YD) independently searched PubMed, Web of Science, the Cochrane Library, ClinicalTrials.gov, and Embase from inception to July 4, 2026, without date or language restrictions, and independently screened titles/abstracts and full texts. The search combined calprotectin/S100-family terms with infection, bacterial infection, sepsis, septic shock, and infection-source terms. Reference lists were also screened, and disagreements were resolved by a third reviewer (GC). Complete search strategies are provided in **Supplementary File 2**.

### Data extraction

Two reviewers (YL and YD) independently extracted study characteristics and diagnostic or prognostic performance data using a standardized form. AUCs were extracted only when reported in the article or **Supplementary Material**; when their uncertainty was unavailable, standard errors and 95% confidence intervals were estimated using the Hanley–McNeil approximation. Threshold-based 2×2 counts were extracted directly or reconstructed from group sizes and reported sensitivity and specificity when internally consistent after allowing for rounding. To avoid double counting, one clinically prioritized endpoint and one author-reported threshold per study and outcome were selected for each primary synthesis. When multiple reports used the same cohort and biomarker endpoint, the estimate was entered only once in a given synthesis; secondary reports were retained only for unique analytes or outcomes. Comparator and combined-model data were extracted separately. Disagreements were resolved by consensus or consultation with a third reviewer (GC). Detailed extraction variables and endpoint-selection rules are provided in **Supplementary File 4**.

### Risk of bias and certainty of evidence

Risk of bias and certainty of evidence were independently assessed by two reviewers (YL and YD). Diagnostic studies were evaluated using the Quality Assessment of Diagnostic Accuracy Studies-2 (QUADAS-2) tool, and prognostic studies received a supplementary assessment covering participant selection, predictor and outcome measurement, and confounding or modeling. Certainty was evaluated using the Grading of Recommendations Assessment, Development and Evaluation (GRADE) framework across risk of bias, inconsistency, indirectness, imprecision, and publication bias domains (Whiting et al., 2011; Schunemann et al., 2008). Disagreements were resolved by consensus, with adjudication by a third reviewer (GC) when necessary.

### Statistical analysis

All analyses and figures were generated using R version 4.6.0 (R Foundation for Statistical Computing, Vienna, Austria). Diagnostic AUCs and directly reported prognostic AUCs were synthesized separately using random-effects models on the logit scale and back-transformed with 95% confidence intervals. Heterogeneity was assessed using I^2^. The main diagnostic AUC analysis included clinically relevant comparator studies and was summarized overall and by target condition: sepsis diagnosis versus acute infection or infection-source-related diagnosis.

Threshold-based sensitivity and specificity were jointly synthesized using bivariate random-effects models (Reitsma approach), with summary receiver operating characteristic plots displaying study-level operating points and summary estimates. Diagnostic and prognostic outcomes were analyzed separately. Exploratory subgroup and stability analyses evaluated study-level characteristics and the influence of broader study designs, study groups, and individual studies; these analyses were not interpreted as formal tests of interaction.

External single-biomarker AUCs were analyzed separately for diagnostic and prognostic outcomes. Within-study differences were calculated as the comparator AUC minus the calprotectin/S100A8/A9 AUC and pooled when at least two studies reported the same comparison. Because paired covariance was rarely reported, the corresponding P values were considered supportive rather than definitive. Combined-model analyses were descriptive and summarized changes in AUC after adding calprotectin/S100A8/A9 to an existing marker or model. Detailed model specifications, subgroup definitions, stability analyses, graphical procedures, AUC-difference calculations, and ROC-reconstruction methods are provided in **Supplementary File 4**.

## Results

### Study selection and characteristics

The search identified 7,964 records. After duplicate removal and screening, 19 reports representing 18 independent cohorts were included from database searches, and three additional studies were identified from the previous meta-analysis, yielding 22 reports representing 21 independent cohorts (**Figure 1**).

**Figure 1:**
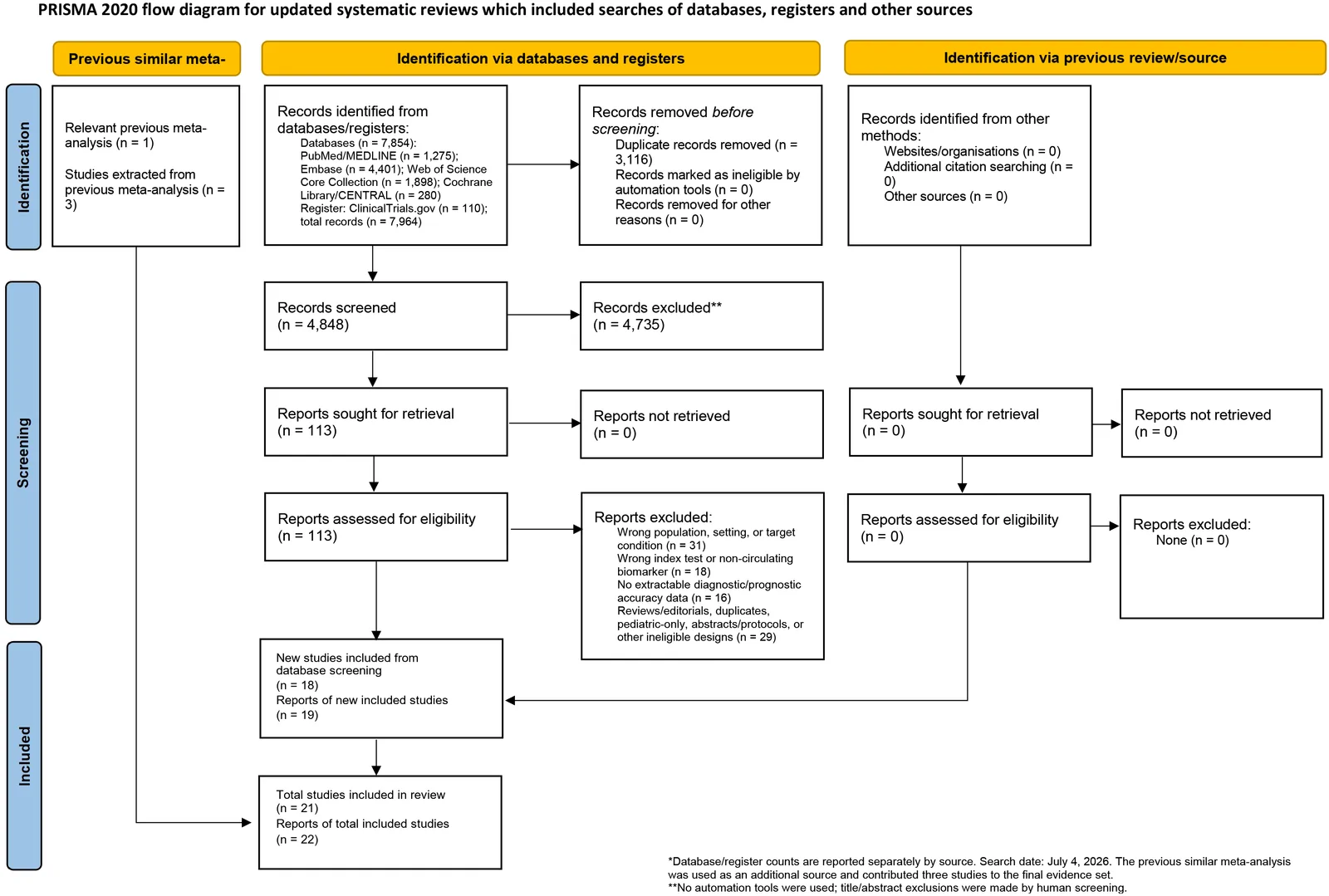
PRISMA flow diagram. Flow of records through identification, screening, eligibility assessment, and inclusion.

Included cohorts spanned emergency, acute-care, intensive-care, respiratory-infection, infection-source, postoperative, and sepsis prognostic settings across Europe and Asia. They evaluated circulating calprotectin or S100A8/A9-related analytes using varied specimens, assays, reference standards, and outcomes (**Supplementary Table 1**). Reports assigned to the exploratory or prognostic evidence sets addressed special comparators, pathogen- or site-specific outcomes, severity-related endpoints, or mortality/survival outcomes (Gao et al., 2015; Huang et al., 2016; Simm et al., 2016; Havelka et al., 2020; Liu et al., 2021; Venge et al., 2021; Garcia de Guadiana-Romualdo et al., 2024; Chen et al., 2025; Gigliotti et al., 2025; Hu et al., 2025; Strambi et al., 2026).

Larsson et al. (2020) and Sejersen et al. (2025b) reported diagnostic and prognostic AUCs for total calprotectin using the same 271-patient ICU cohort. To avoid double counting, total-calprotectin estimates were included only once in each synthesis, whereas the S100A8 and S100A9 subunit results reported by Sejersen et al. were retained because they provided distinct information.

After accounting for overlapping cohort reports, 10 independent evidence records contributed to the prespecified main diagnostic AUC synthesis, 19 to the broader exploratory AUC synthesis, 10 provided diagnostic 2×2 data, including 5 main-analysis and 5 exploratory studies, and 6 contributed mortality/survival threshold-based analyses. The study-level composition of each evidence set is provided in **Supplementary Table 1**.

### Risk of bias and certainty of evidence

Risk of bias varied across studies, and certainty of evidence was very low for all major diagnostic and prognostic outcomes. Detailed QUADAS-2 judgments and GRADE assessments are provided in **Supplementary Tables 2** and **3**.

### Main diagnostic AUC synthesis

In the descriptive main diagnostic AUC synthesis of 10 independent evidence records, the pooled AUC was 0.741 (95% CI, 0.657–0.810; I² = 94.6%) (**Figure 2**).

**Figure 2:**
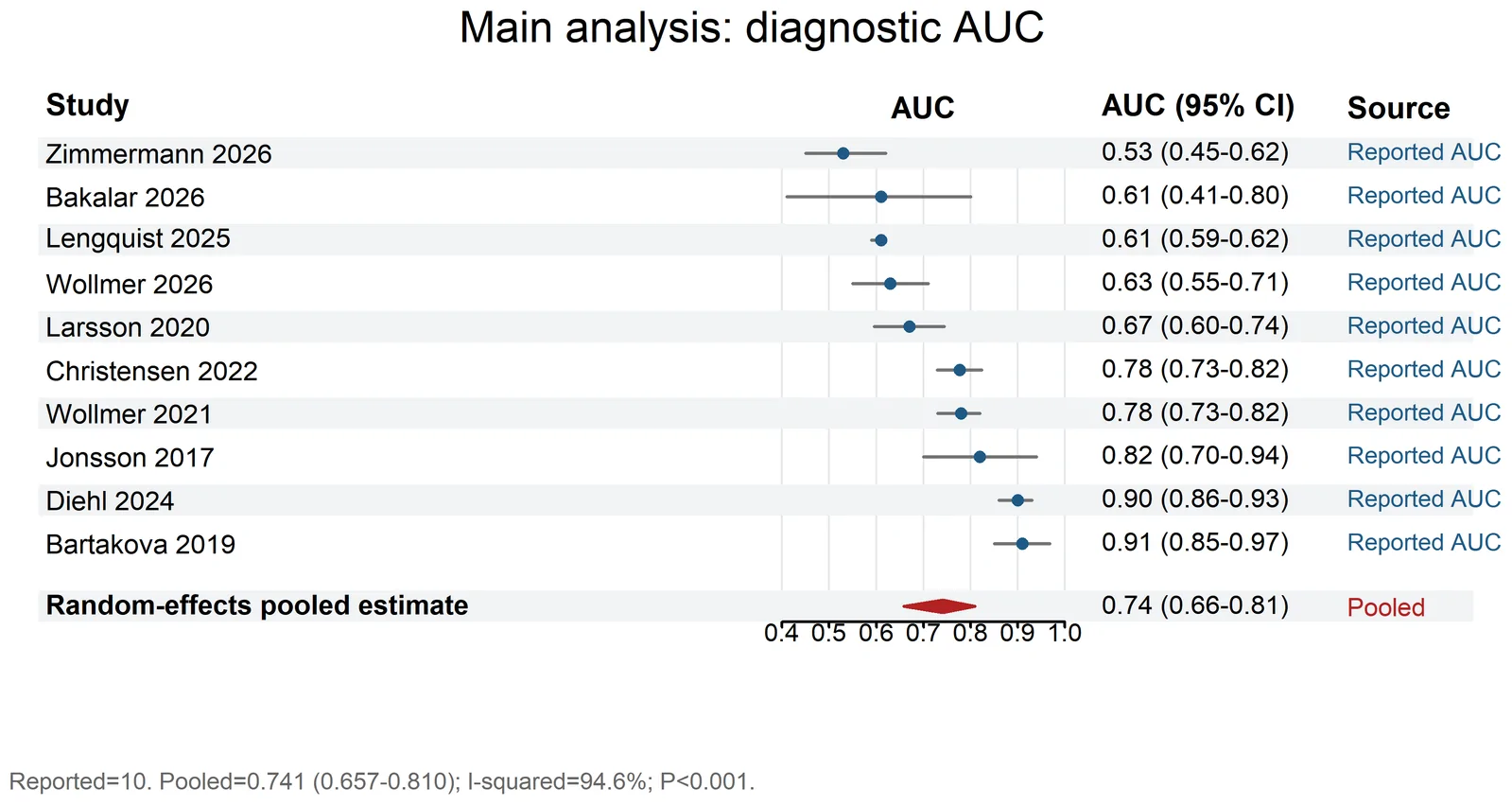
Main diagnostic AUC synthesis. Random-effects forest plot of AUCs from the main clinical-comparator evidence set after counting overlapping cohort estimates once.

Four independent cohorts evaluated sepsis diagnosis and yielded a pooled AUC of 0.703 (95% CI, 0.600–0.789; I² = 89.3%), whereas six evaluated acute infection or infection-source-related outcomes and yielded a pooled AUC of 0.758 (95% CI, 0.636–0.849; I² = 92.6%) (**Figure 3**). This numerical difference was exploratory and was not tested as a formal between-subgroup interaction.

**Figure 3:**
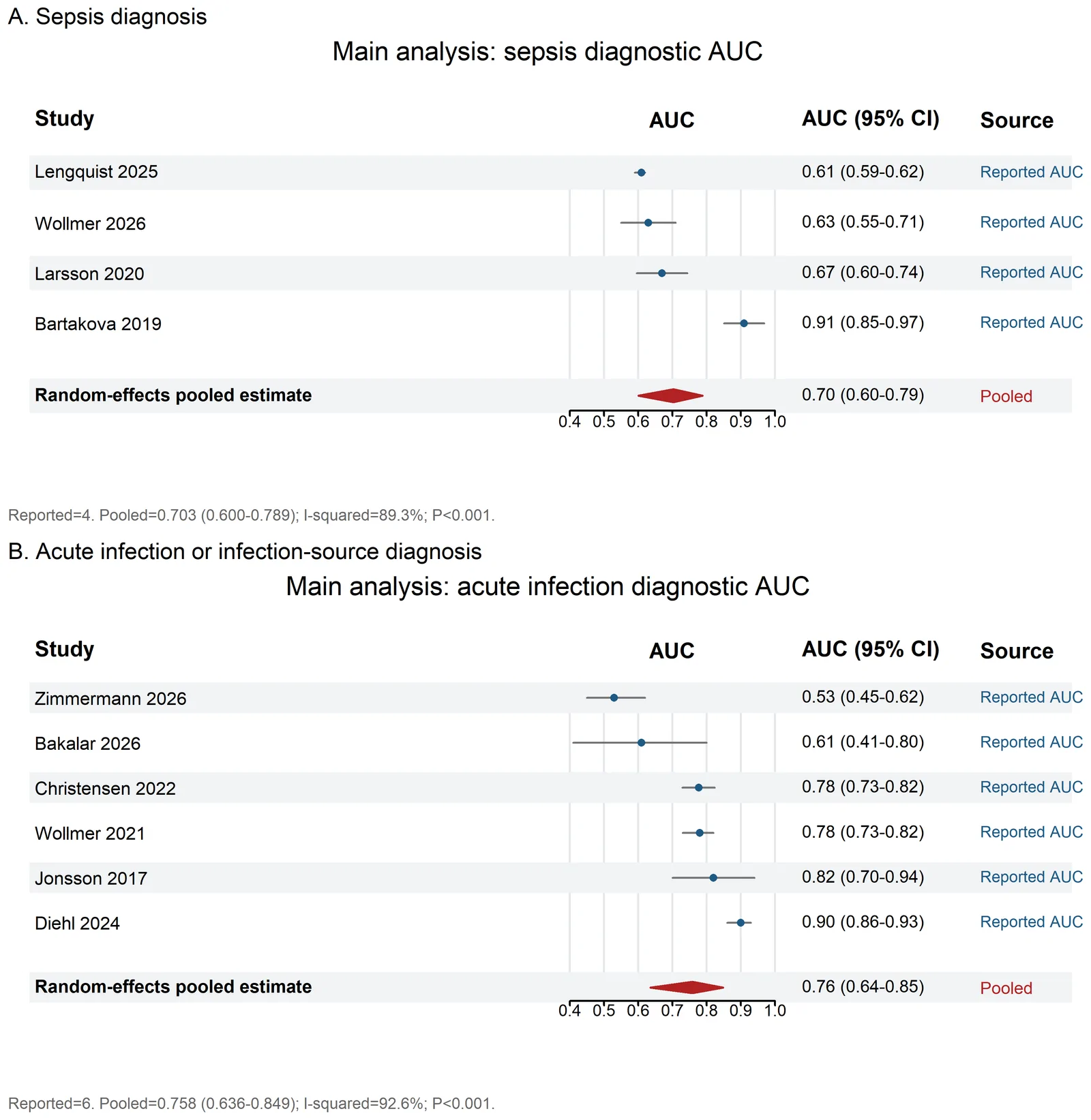
Target-specific diagnostic AUC synthesis. Forest plots of AUCs for sepsis diagnosis and acute infection or infection-source-related diagnosis.

### Exploratory diagnostic subgroup analyses

Exploratory subgroup estimates varied according to the availability of 2×2 data, clinical setting, and reference standard, but these differences were not tested as formal interactions and may have been confounded by target condition and study design (**Supplementary Figure S1**).

### Threshold-based diagnostic performance

Five studies met the criteria for main threshold-based 2×2 pooling: two for sepsis diagnosis and three for acute infection or source-related diagnosis. Pooled sensitivity and specificity were 0.70 (95% CI, 0.46–0.86) and 0.57 (95% CI, 0.55–0.59), respectively, for sepsis, and 0.46 (95% CI, 0.09–0.88) and 0.86 (95% CI, 0.60–0.96), respectively, for acute infection/source-related outcomes (**Figure 4**; **Supplementary Figures S2**-**S4**).

**Figure 4:**
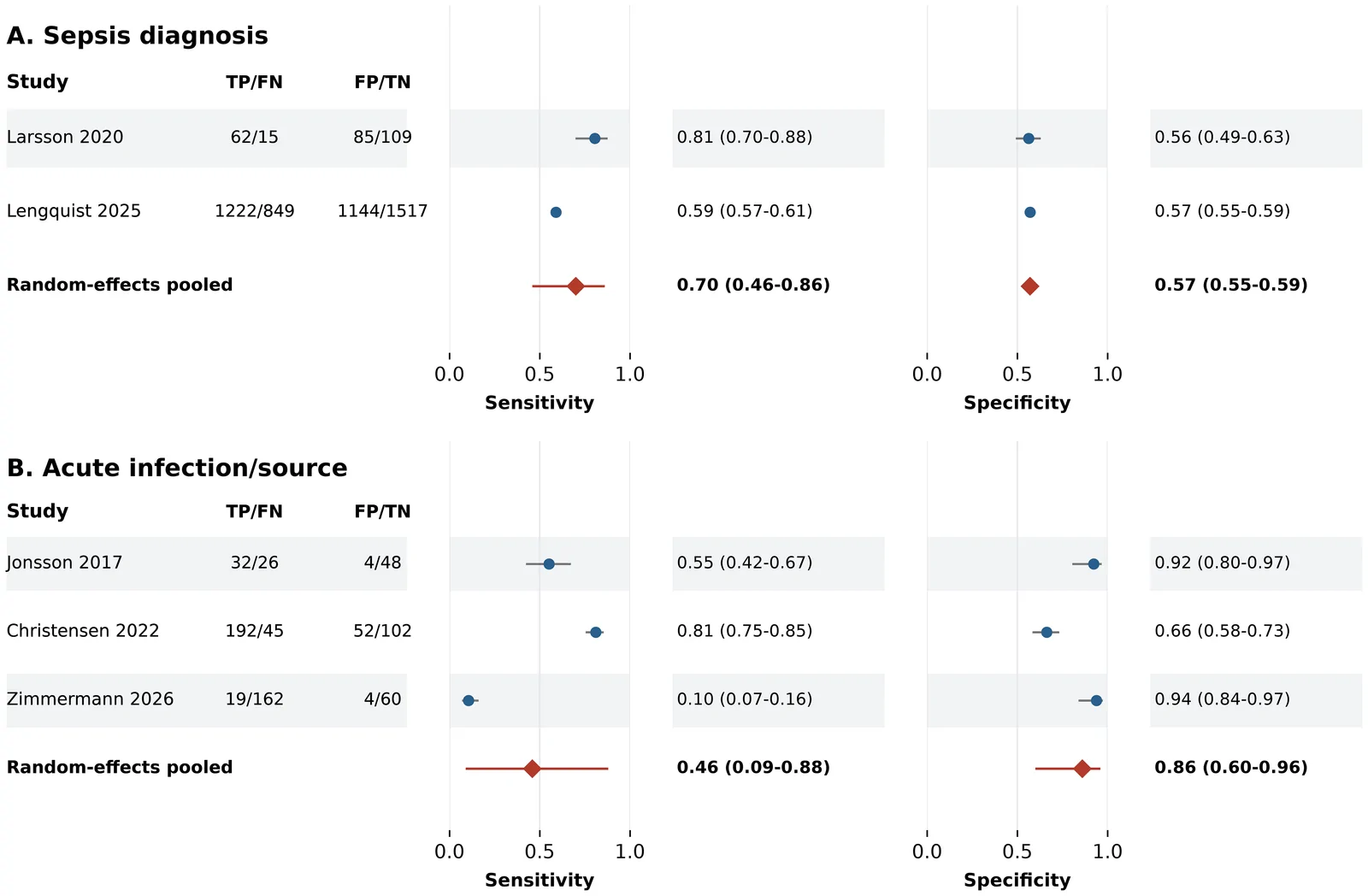
Main threshold-based diagnostic performance. Forest plots of sensitivity and specificity for sepsis diagnosis and infection/source-related diagnosis based on main-analysis 2×2 studies.

Exploratory threshold-based subgroup estimates varied across cut-off and clinical-setting categories (**Figure 5**). These descriptive findings were not interpreted as formal interaction tests.

**Figure 5:**
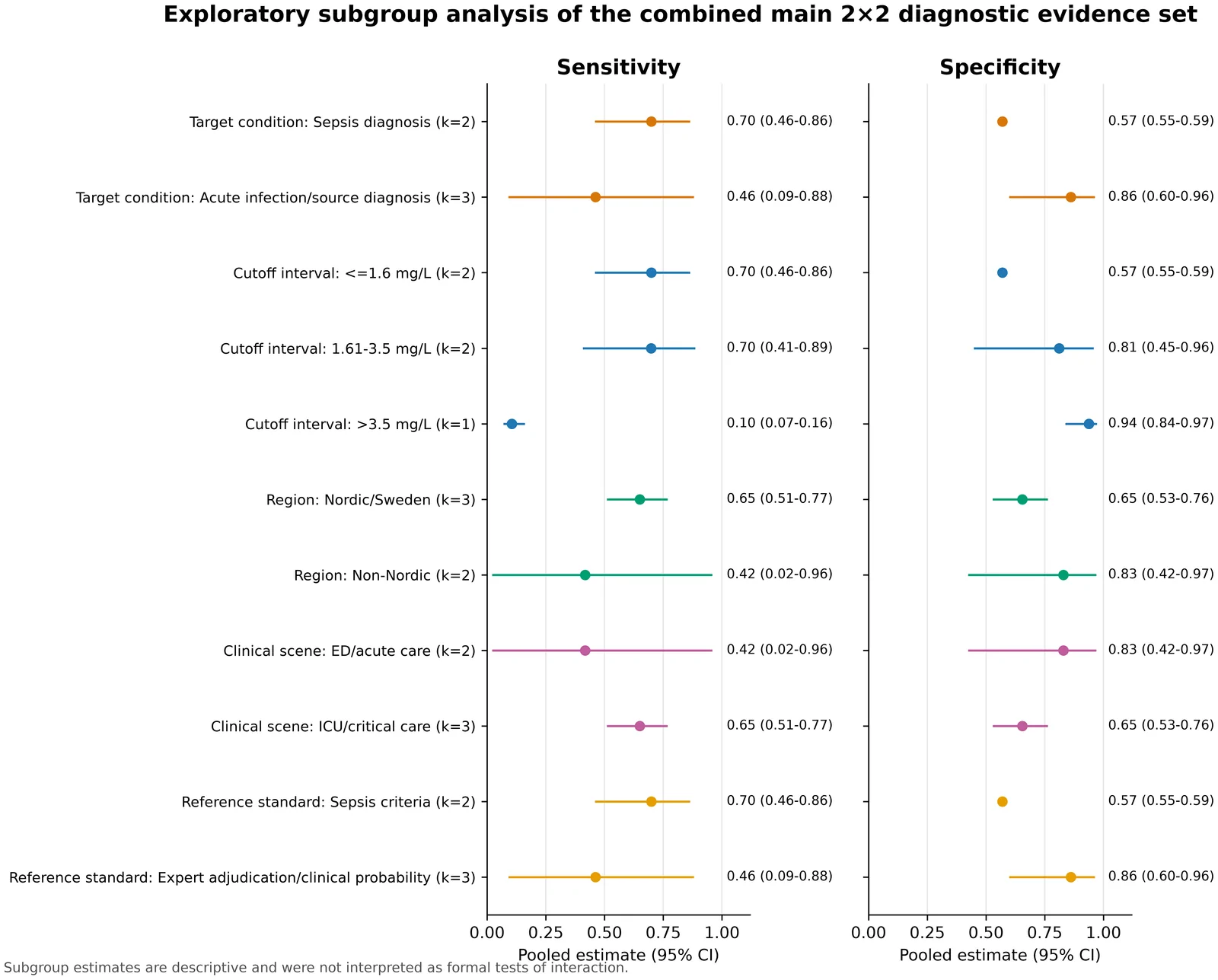
Exploratory subgroup analysis of the main threshold-based diagnostic evidence set. Descriptive sensitivity and specificity summaries for the combined main 2×2 evidence set; these subgroup estimates were not interpreted as formal tests of interaction.

### Exploratory broader diagnostic analyses

In the broader exploratory evidence set, the pooled AUC across 19 independent evidence records was 0.794 (95% CI, 0.735–0.843; I² = 93.9%). AUC stability analyses using sequential addition of prespecified design groups, leave-one-design-group-out analyses, and leave-one-study-out analyses did not materially change the direction of the pooled estimate despite substantial heterogeneity. In a descriptive 10-study 2×2 pool combining heterogeneous targets and thresholds, summary sensitivity was 0.66 (95% CI, 0.52–0.78) and specificity was 0.77 (95% CI, 0.68–0.84). Deeks’ test suggested possible small-study effects (P = 0.024) (Deeks et al., 2005) (**Supplementary Figures S5**-**S8**).

### Comparison with other diagnostic biomarkers

Separate pooled AUCs are shown in **Supplementary Figure S9**. Supportive within-study comparisons suggested a higher AUC for CRP than for calprotectin/S100A8/A9 and a lower AUC for WBC, with no clear difference for PCT. Because paired covariance was unavailable and standard errors were approximated, these analyses did not establish biomarker superiority.

Across five endpoints from three studies, adding calprotectin was associated with small AUC increases for selected emergency-department or infection-source outcomes, but no increase was observed when it was added to a model containing body temperature, WBC, and CRP. No formal pooled incremental-effect estimate was calculated (**Supplementary Figure S9**).

### Prognostic and severity-related outcomes

Six studies contributed threshold-based mortality/survival analyses, with pooled sensitivity of 0.72 (95% CI, 0.65–0.77) and specificity of 0.66 (95% CI, 0.53–0.77). The forest plot is shown in **Figure 6**, with supporting analyses in **Supplementary Figure S10**. Eight independent cohorts contributed reported prognostic AUCs, yielding a pooled AUC of 0.737 (95% CI, 0.688–0.786; I² = 63.6%). The single eligible prognostic combined-model study reported identical AUCs with and without calprotectin added to SOFA (0.736 vs. 0.736), precluding quantitative synthesis. Threshold-effect analysis suggested cut-off dependence, whereas Deeks’ test did not show clear small-study effects; both assessments were limited by the small and heterogeneous evidence set (**Supplementary Figure S10**).

**Figure 6:**
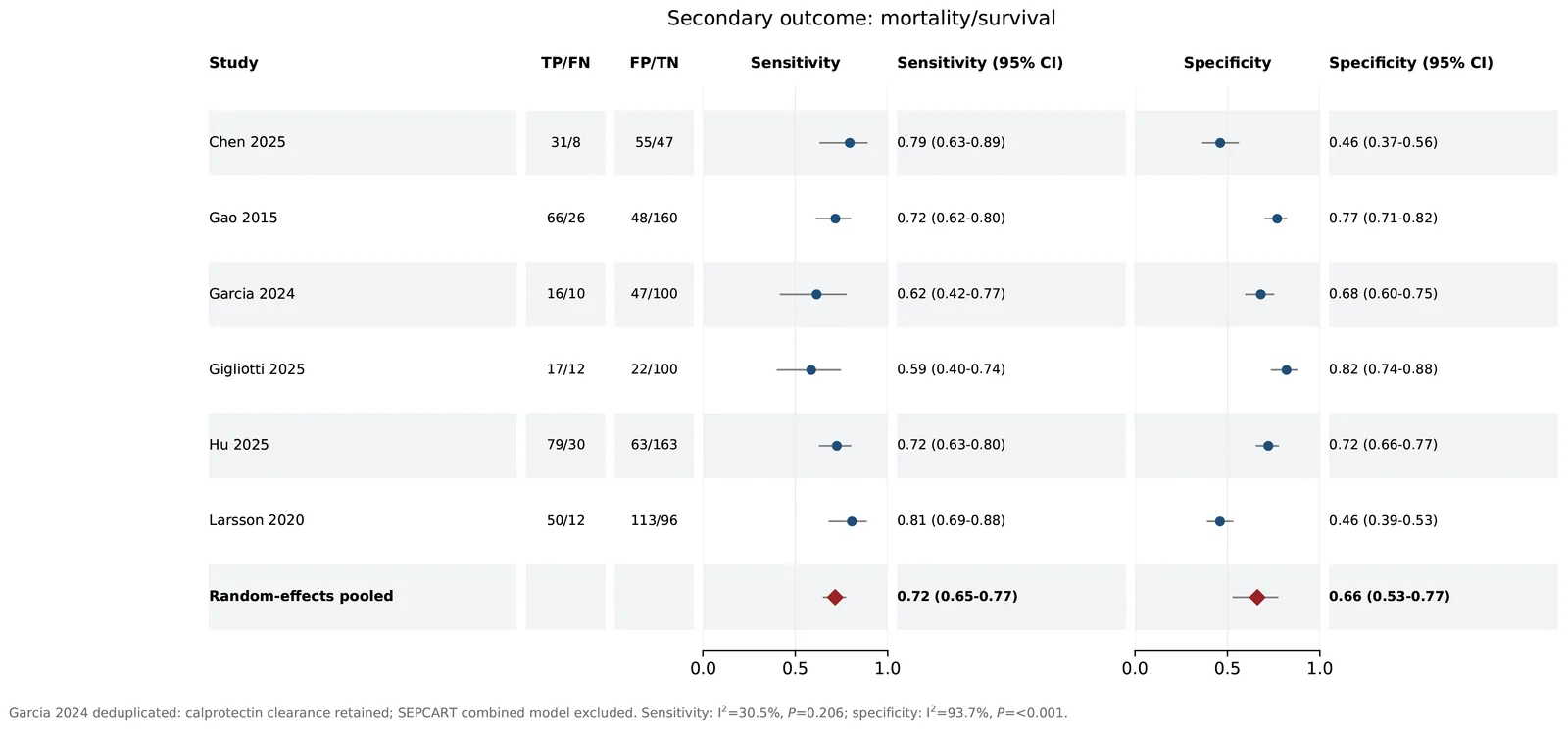
Mortality/survival threshold-based performance. Forest plot of sensitivity and specificity for mortality or survival outcomes.

### Comparison with other prognostic biomarkers

Four studies contributed prognostic single-biomarker comparator data (**Supplementary Table 1**; **Supplementary Figure S11**). Supportive paired analysis yielded a small AUC difference between SOFA and calprotectin/S100A8/A9 (difference 0.090; 95% CI, 0.002–0.178; P = 0.045), whereas comparisons with PCT, CRP, and lactate showed no clear differences. Given the limited number of studies, unavailable paired covariance, and approximated standard errors, this supportive result did not establish the superiority of SOFA.

## Discussion

### Principal findings

This systematic review provides a clinically conservative reassessment of circulating calprotectin and S100A8/A9-related biomarkers in adults with suspected acute infection or sepsis. These biomarkers provided moderate, context-dependent diagnostic information, with substantial heterogeneity and very low certainty of evidence. AUC estimates were numerically more favorable for acute infection or infection-source-related outcomes than for broad sepsis diagnosis, but this contrast was hypothesis-generating, and the threshold-based and prognostic findings were exploratory. Overall, calprotectin-related biomarkers appear more suitable as adjunctive host-response markers than as stand-alone diagnostic or prognostic tests.

### Comparison with previous reviews and current biomarker research

These findings refine rather than directly contradict earlier evidence. A previous meta-analysis reported favorable diagnostic accuracy for ICU sepsis but searched only through January 2022, included few records, and combined adult and neonatal evidence (Gao et al., 2022). By focusing the main interpretation on clinically realistic adult comparator studies, separating diagnostic from prognostic outcomes, and analyzing special-control or site-specific studies as exploratory evidence, the present review provides a more cautious and clinically relevant estimate. Its added value lies not only in including additional studies but also in reorganizing the evidence around the question faced by clinicians evaluating undifferentiated suspected infection or sepsis.

Recent reviews indicate that sepsis biomarker research has moved beyond the search for a single universal marker. Established markers such as C-reactive protein and procalcitonin remain widely used, while emerging host-response markers, including calprotectin/S100A8/A9, presepsin, heparin-binding protein, monocyte distribution width, and selected cytokine, endothelial, and cardiac markers, are being evaluated for early recognition, antibiotic guidance, circulatory support, and immune-targeted treatment (Bourika et al., 2025; Sejersen et al., 2025a; Wang et al., 2023). However, most remain context-dependent and lack standardized thresholds, external validation, or evidence that biomarker-guided use improves patient-centered outcomes. Current research therefore increasingly combines serial biomarker trajectories, multimarker or multimodal models, rapid microbiological diagnostics, and biomarker-based enrichment of clinical trials (Van Nynatten et al., 2025; Wester et al., 2026; Zheng et al., 2026). Consistent with this broader perspective, our exploratory diagnostic and prognostic biomarker comparisons, together with the limited combined-model analyses, support evaluating calprotectin as a context-dependent component of multimarker assessment rather than as a replacement for established biomarkers or clinical models. However, heterogeneity in endpoints, limited comparator evidence, unavailable paired covariance, and the descriptive nature of the model analyses preclude firm conclusions about biomarker superiority or incremental clinical utility. Nevertheless, from a biological perspective, there remains a rationale for investigating calprotectin as a host-response biomarker because its release is linked to myeloid activation and pathways involved in tissue and organ injury. Recent mechanistic studies of ARDS and bacterial lung injury have linked FKBP5-mediated necroptosis to neutrophil recruitment and lung injury and implicated heparan-sulfate/glycocalyx-related signaling and STAT3-dependent endothelial-barrier regulation in LPS-related lung injury (Cui et al., 2026; Zhang et al., 2025, 2026a, b). Although these studies did not directly evaluate circulating calprotectin or S100A8/A9, they provide mechanistic context for investigating myeloid-activation biomarkers but do not establish their diagnostic accuracy, prognostic value, or clinical utility.

### Why broad sepsis diagnosis is a difficult target

Broad sepsis diagnosis is challenging for any circulating biomarker because sepsis is a syndrome of infection-associated organ dysfunction rather than a microbiological diagnosis. Increased myeloid activation cannot establish that infection caused the inflammation or organ dysfunction. Sepsis classification requires attribution: clinicians must determine whether organ dysfunction is caused by infection and whether competing inflammatory explanations are present. In emergency and critical-care populations, surgery, trauma, burns, ischemia, malignancy, autoimmune disease, immunosuppression, renal dysfunction, mechanical ventilation, and recent antibiotics can all alter inflammatory biomarkers and blur the boundary between infectious and sterile inflammation. This clinical heterogeneity may explain the less favorable performance of calprotectin-related biomarkers for broad sepsis classification than for more specific infection-related contrasts. Findings from larger, clinically heterogeneous cohorts therefore support an adjunctive rather than stand-alone role in unselected critically ill adults.

### Interpretation of infection-source and bacterial-infection contrasts

The numerically more favorable estimates for acute infection or source-related outcomes are biologically plausible because bacterial and lower respiratory tract infections often involve neutrophil-dominant inflammation. Calprotectin may therefore capture a host-response dimension that differs from hepatic acute-phase proteins and PCT. However, biological coherence should not be confused with clinical proof. Pathogen- or source-specific studies commonly use cleaner contrasts than those encountered at clinical presentation; for example, a comparison between bacterial pneumonia and viral respiratory infection is not equivalent to undifferentiated dyspnea in an emergency department patient with heart failure, chronic lung disease, malignancy, immunosuppression, or prior antibiotic exposure. Healthy, elective-surgical, or other special controls may also increase biological separation between groups. Such studies support biological interpretation but should not determine the main estimate of performance in undifferentiated clinical populations.

### Strengths and limitations

This review separated clinically realistic comparator studies from studies with special designs, analyzed AUC and threshold-based data separately, distinguished diagnostic from prognostic outcomes, and evaluated exploratory comparisons with established biomarkers and SOFA. These choices supported a more conservative and clinically relevant interpretation.

Nevertheless, heterogeneity was substantial, only five main-analysis studies provided threshold-based 2×2 data, and target conditions and reference standards varied. Many AUC comparisons lacked the covariance required for formal paired testing, comparator evidence was often based on only one or two studies, and prognostic studies differed in outcome horizons and confounding adjustment. Publication bias or small-study effects could not be excluded. Analytical heterogeneity in analyte definition, specimen type, assay platform and calibration, sampling time, and pre-analytical handling also limits measurement interchangeability and cut-off transferability (Sejersen et al., 2025a). Consequently, certainty was very low across the major evidence bodies.

## Conclusions

Circulating calprotectin and S100A8/A9-related biomarkers provide moderate, context-dependent diagnostic information in adults with suspected acute infection or sepsis. Numerically more favorable AUC estimates for acute infection or infection-source-related outcomes than for broad sepsis diagnosis were hypothesis-generating; substantial heterogeneity, inconsistent thresholds, limited threshold-based data, and very low certainty do not support stand-alone use or universal cut-offs. Exploratory head-to-head comparisons showed differences in AUC estimates but did not establish the superiority of calprotectin or any comparator biomarker. Calprotectin-related biomarkers should therefore be considered potential adjuncts to clinical assessment and established laboratory and severity measures rather than replacements for them. Further studies should determine their incremental value using standardized assays, prespecified models, and clinically relevant outcomes.

## Data Availability

The original contributions presented in the study are included in the article/**Supplementary Material**. Further inquiries can be directed to the corresponding author/s.

## Supplementary material

Supplementary Figure 1 Exploratory subgroup analyses of the main diagnostic AUC synthesis by target condition, availability of 2×2 data, clinical setting, and reference standard. Single-study strata were not interpreted as pooled evidence.

Supplementary Figure 2 Main diagnostic SROC plots and likelihood-ratio scattergrams. **(A)** SROC plot for sepsis diagnosis. **(B)** SROC plot for acute infection or infection-source-related diagnosis. **(C)** Likelihood-ratio scattergram for sepsis diagnosis. **(D)** Likelihood-ratio scattergram for acute infection or infection-source-related diagnosis.

Supplementary Figure 3 Main diagnostic post-test probability and threshold-effect analyses. **(A)** Fagan-style plot for sepsis diagnosis. **(B)** Fagan-style plot for acute infection or infection-source-related diagnosis. **(C)** Threshold-effect plot for sepsis diagnosis. **(D)** Threshold-effect plot for acute infection or infection-source-related diagnosis.

Supplementary Figure 4 Main diagnostic heterogeneity and small-study-effect analyses. **(A)** Galbraith plot for sepsis diagnosis. **(B)** Galbraith plot for acute infection or infection-source-related diagnosis. **(C)** Deeks’ funnel plot for sepsis diagnosis. **(D)** Deeks’ funnel plot for acute infection or infection-source-related diagnosis.

Supplementary Figure 5 Broader exploratory diagnostic AUC synthesis including the main clinical-comparator studies and exploratory or sensitivity-analysis studies.

Supplementary Figure 6 Broader exploratory threshold-based diagnostic analyses. **(A)** Forest plot of sensitivity and specificity. **(B)** Corresponding summary receiver operating characteristic display.

Supplementary Figure 7 Broader exploratory threshold-effect and small-study-effect analyses. **(A)** Threshold-effect plot. **(B)** Deeks’ funnel plot.

Supplementary Figure 8 Exploratory diagnostic AUC stability analyses. **(A)** Sequential addition of exploratory design categories. **(B)** Leave-one-design-group-out analysis. **(C)** Leave-one-study-out analysis.

Supplementary Figure 9 Exploratory diagnostic single-biomarker and combined-model analyses. The upper section shows pooled diagnostic AUC displays and AUC differences versus calprotectin/S100A8/A9; the lower section shows reconstructed ROC curves and endpoint-level incremental changes for combined models. Reconstructed curves are visual summaries rather than patient-level SROC curves.

Supplementary Figure 10 Mortality/survival threshold-performance supporting analyses. **(A)** SROC plot. **(B)** Fagan-style post-test probability plot. **(C)** Threshold-effect plot. **(D)** Deeks’ funnel plot.

Supplementary Figure 11 Prognostic analyses. The upper section shows the prognostic AUC synthesis for mortality/survival outcomes based on eight independent cohorts with directly reported ROC AUCs. The lower section shows prognostic single-biomarker comparisons; AUC differences were calculated as comparator minus calprotectin/S100A8-A9 and interpreted as supportive because paired covariance was generally unavailable.

Supplementary Table 1 Characteristics of included studies. Country, clinical setting, design, population, target condition, analyte, specimen, assay, reference standard, sample size, and analysis role.

Supplementary Table 2 QUADAS-2 risk-of-bias and applicability assessment for diagnostic studies, with supplementary prognostic risk-of-bias notes where applicable.

Supplementary Table 3 GRADE certainty of evidence by evidence body.

Supplementary File 1 Completed PRISMA-DTA checklist with the reporting location for each checklist item.

Supplementary File 2 Complete database search strategies.

Supplementary File 3 Supplementary tables, including **Supplementary Table 1** (characteristics of included studies), **Supplementary Table 2** (QUADAS-2 risk-of-bias and applicability assessments), and **Supplementary Table 3** (GRADE certainty of evidence by evidence body).

Supplementary File 4 Supplementary methods, including detailed eligibility criteria, evidence-set definitions, data-extraction and endpoint-selection rules, and statistical analyses.
