# Supplementary material for: Circulating calprotectin and S100A8/A9-related biomarkers for diagnosis and prognostic stratification in adult acute infection and sepsis: a systematic review and meta-analysis
Source: Front Cell Infect Microbiol. 2026 Oct 14;16:1934020. doi: 10.3389/fcimb.2026.1934020 (PMC13713635; doi:10.3389/fcimb.2026.1934020)
Supplement: Supplementary file 2 [file Supplementaryfile2.docx]

Supplementary File 2. Detailed Database Search Strategies

Search date: July 4, 2026. Databases searched from inception to July 4, 2026 were PubMed/MEDLINE, Embase, Web of Science Core Collection, the Cochrane Library/CENTRAL, and ClinicalTrials.gov. Scopus and the World Health Organization International Clinical Trials Registry Platform were not searched.

**Search concept used in the manuscript:** The bibliographic search strategy combined terms for calprotectin and related S100-family analytes with terms for infection, bacterial infection, sepsis, septic shock, and related acute infectious presentations. A deliberately broad analyte-plus-condition strategy was used to maximize sensitivity and reduce the risk of missing clinically relevant diagnostic or prognostic biomarker studies that did not explicitly report performance terms in the title, abstract, or keywords. Diagnostic and prognostic relevance was therefore assessed during study screening and full-text review rather than by a restrictive third search block. No date or language restrictions were applied at the search-design stage. Reference lists of included studies and relevant reviews were also screened for additional eligible records. The full database-specific search strategies are provided below.

**PubMed/MEDLINE (N = 1,275)**

((calprotectin[tiab] OR "S100A8/A9"[tiab] OR "S100A8-A9"[tiab] OR S100A8[tiab] OR S100A9[tiab] OR "MRP8/14"[tiab] OR MRP8[tiab] OR MRP14[tiab] OR "myeloid-related protein 8/14"[tiab] OR "leukocyte L1 antigen"[tiab] OR "calgranulin A"[tiab] OR "calgranulin B"[tiab])) AND (("Sepsis"[Mesh] OR "Shock, Septic"[Mesh] OR sepsis[tiab] OR "septic shock"[tiab] OR infection[tiab] OR "bacterial infection"[tiab] OR "systemic inflammatory response syndrome"[tiab] OR SIRS[tiab] OR pneumonia[tiab] OR "lower respiratory tract infection"[tiab] OR bacteremia[tiab] OR bacteraemia[tiab] OR "bloodstream infection"[tiab] OR "infection source"[tiab]))

**Embase (N = 4,401)**

('calprotectin'/exp OR calprotectin:ti,ab,kw OR 's100 calcium binding protein a8'/exp OR s100a8:ti,ab,kw OR 's100 calcium binding protein a9'/exp OR s100a9:ti,ab,kw OR 's100a8/a9':ti,ab,kw OR 's100a8-a9':ti,ab,kw OR 'mrp8/14':ti,ab,kw OR mrp8:ti,ab,kw OR mrp14:ti,ab,kw OR 'myeloid related protein 8/14':ti,ab,kw OR 'leukocyte l1 antigen':ti,ab,kw OR 'calgranulin a':ti,ab,kw OR 'calgranulin b':ti,ab,kw) AND ('bacterial infection'/exp OR sepsis/exp OR 'septic shock'/exp OR pneumonia/exp OR bacteremia/exp OR infection:ti,ab,kw OR 'bacterial infection':ti,ab,kw OR sepsis:ti,ab,kw OR 'septic shock':ti,ab,kw OR 'systemic inflammatory response syndrome':ti,ab,kw OR sirs:ti,ab,kw OR pneumonia:ti,ab,kw OR 'lower respiratory tract infection':ti,ab,kw OR bacteremia:ti,ab,kw OR bacteraemia:ti,ab,kw OR 'bloodstream infection':ti,ab,kw OR 'infection source':ti,ab,kw)

**Web of Science Core Collection (N = 1,898)**

TS=(calprotectin OR "S100A8/A9" OR "S100A8-A9" OR S100A8 OR S100A9 OR "MRP8/14" OR MRP8 OR MRP14 OR "myeloid-related protein 8/14" OR "leukocyte L1 antigen" OR "calgranulin A" OR "calgranulin B") AND TS=(infection OR "bacterial infection" OR sepsis OR "septic shock" OR "systemic inflammatory response syndrome" OR SIRS OR pneumonia OR "lower respiratory tract infection" OR bacteremia OR bacteraemia OR "bloodstream infection" OR "infection source")

**Cochrane Library/CENTRAL (N = 280)**

(calprotectin OR "S100A8/A9" OR "S100A8-A9" OR S100A8 OR S100A9 OR "MRP8/14" OR MRP8 OR MRP14 OR "myeloid-related protein 8/14" OR "leukocyte L1 antigen" OR "calgranulin A" OR "calgranulin B") AND (infection OR "bacterial infection" OR sepsis OR "septic shock" OR "systemic inflammatory response syndrome" OR SIRS OR pneumonia OR "lower respiratory tract infection" OR bacteremia OR bacteraemia OR "bloodstream infection" OR "infection source")

**ClinicalTrials.gov (N = 110)**

Condition or disease: infection OR "bacterial infection" OR sepsis OR "septic shock" OR pneumonia OR bacteremia OR "bloodstream infection"; Other terms: calprotectin OR "S100A8/A9" OR S100A8 OR S100A9 OR "MRP8/14"; Intervention/treatment: not used.
