# Supplementary material for: Circulating calprotectin and S100A8/A9-related biomarkers for diagnosis and prognostic stratification in adult acute infection and sepsis: a systematic review and meta-analysis
Source: Front Cell Infect Microbiol. 2026 Oct 14;16:1934020. doi: 10.3389/fcimb.2026.1934020 (PMC13713635; doi:10.3389/fcimb.2026.1934020)
Supplement: Supplementary file 3 [file Supplementaryfile3.docx]

Supplementary File 3. Supplementary Tables

**Supplementary Table 1. Characteristics of included studies.**

| **Study** | **Country/region** | **Analysis role in final version** | **Primary diagnostic/prognostic endpoint used** | **Target condition group** | **Clinical setting** | **Study design/group** | **Reference standard/outcome definition** | **Analyte** | **Specimen** | **Assay** | **Approximate analysed N** | **Preferred AUC used in final diagnostic/prognostic synthesis** | **Main-analysis eligibility note** |
| --- | --- | --- | --- | --- | --- | --- | --- | --- | --- | --- | --- | --- | --- |
| Bakalar et al., 2026 | Czech Republic; Europe | Main diagnostic AUC | Infected vs non-infected burn ICU patients | Acute infection/source diagnosis | Burn ICU/critical care | Prospective single-center observational study | Composite clinical infection reference standard using microbiology, imaging when available, and antimicrobial-treatment decisions | Circulating/blood calprotectin | Blood; serum/plasma type not specified | QUANTA Flash Circulating Calprotectin electrochemiluminescent assay, Inova Diagnostics/Cobas Roche | 60 | 0.61 (0.41–0.80) | Eligible for main analysis under clinical suspected infection/sepsis comparator principle. |
| Bartakova et al., 2019 | Czech Republic; Europe | Main diagnostic AUC | Bacterial sepsis vs confirmed viral infection | Sepsis diagnosis | Department of Infectious Diseases/acute clinic; hospitalized bacterial sepsis and viral-infection patients plus healthy controls | Prospective single-center observational study | Bacterial sepsis by Levy 2003 sepsis criteria plus clinical/laboratory/radiologic infection evidence; viral infection confirmed by serology/PCR/antigen | Serum calprotectin (S100A8/A9) and calgranulin C/S100A12 | Serum | ELISA, BioVendor kits on EVOLIS microplate system | 90 for preferred BS vs viral endpoint; 116 total including healthy controls | 0.91 (0.85–0.97) | Eligible for main analysis under clinical suspected infection/sepsis comparator principle. |
| Chen et al., 2025 | China; Asia | Secondary mortality/survival AUC; Secondary mortality/survival 2×2 | 28-day mortality | Mortality/survival prognosis | Sepsis/critical illness prognosis | Observational/prognostic | 28-day mortality | Calprotectin/S100A8/A9-related | Blood/plasma/serum (as reported) | As reported | 141 | Prognostic mortality/survival AUC: 0.62 (0.51–0.72), reported ROC AUC; threshold-based mortality/survival analysis used 2×2 data | Secondary prognostic outcome; not part of diagnostic main analysis; contributes to reported-AUC mortality/survival synthesis and mortality/survival 2×2 analysis. |
| Christensen et al., 2022 | Denmark/Europe; Europe | Main diagnostic AUC; Main diagnostic 2×2 | Infection vs not likely infection | Acute infection/source diagnosis | ED/acute care | Prospective/observational | Expert adjudication/clinical probability | Total/blood calprotectin | Plasma/blood | Other/unclear assay | 391 | 0.78 (0.73–0.82) | Eligible for main analysis under clinical suspected infection/sepsis comparator principle. |
| Diehl-Wiesenecker et al., 2024 | Germany; Europe | Main diagnostic AUC; Secondary mortality/survival AUC | Bacterial/co-infection vs viral/no infection | Acute infection/source diagnosis | Emergency department/acute care | Prospective observational study | Expert adjudication for bacterial/viral/no infection; sepsis defined as infection plus >=2 SOFA organ systems within 72 h | Blood calprotectin | Lithium-heparin plasma; subset serum converted to plasma-equivalent | Gentian PETIA/turbidimetric immunoassay on Roche Cobas c501/cobas 8000 | 395 | Diagnostic AUC: 0.90 (0.86–0.93); prognostic mortality/survival AUC: 0.78 (0.71–0.86), reported ROC AUC | Eligible for main diagnostic analysis; also contributes to reported-AUC mortality/survival synthesis. |
| Gao et al., 2015 | China; Asia | Exploratory diagnostic AUC; Exploratory diagnostic 2×2; Secondary mortality/survival AUC; Secondary mortality/survival 2×2 | Sepsis groups vs SIRS plus healthy controls | Sepsis diagnosis | Source-specific/surgical/other | Prospective/observational | Sepsis criteria | S100A8/A9-related | Serum | ELISA/ECL | 353 | Exploratory diagnostic AUC: 0.90 (0.85–0.94); prognostic mortality/survival AUC: 0.81 (0.75–0.88), reported ROC AUC; threshold-based mortality/survival analysis used 2×2 data | Exploratory/sensitivity for diagnosis because of SIRS plus healthy controls; contributes to reported-AUC mortality/survival synthesis and mortality/survival 2×2 analysis. |
| Garcia de Guadiana-Romualdo et al., 2024 | Spain; Europe | Secondary mortality/survival AUC; Secondary mortality/survival 2×2 | 28-day mortality | Mortality/survival prognosis | Sepsis/critical illness prognosis | Observational/prognostic | 28-day mortality | Calprotectin/S100A8/A9-related | Blood/plasma/serum (as reported) | As reported | 173 | Prognostic mortality/survival AUC: 0.77 (0.68–0.86), reported ROC AUC; threshold-based mortality/survival analysis used 2×2 data | Secondary prognostic outcome; not part of diagnostic main analysis; contributes to reported-AUC mortality/survival synthesis and mortality/survival 2×2 analysis. |
| Gigliotti et al., 2025 | Italy/Europe; Europe | Exploratory diagnostic AUC; Exploratory diagnostic 2×2; Secondary mortality/survival AUC; Secondary mortality/survival 2×2 | Sepsis groups vs control/SIRS no bloodstream infection | Sepsis diagnosis | Source-specific/surgical/other | Retrospective/biobank | Microbiology/clinical source diagnosis | Total/blood calprotectin | Serum | ELISA/ECL | 151 | Exploratory diagnostic AUC: 0.86 (0.79–0.92); prognostic mortality/survival AUC: 0.72 (0.63–0.80), reported ROC AUC; threshold-based mortality/survival analysis used 2×2 data | Exploratory/sensitivity for diagnosis because of Gram-negative bloodstream infection/bacteremia-specific design; contributes to reported-AUC mortality/survival synthesis and mortality/survival 2×2 analysis. |
| Havelka et al., 2020 | Czech Republic; Europe | Exploratory diagnostic AUC; Exploratory diagnostic 2×2 | Bacterial pneumonia vs viral respiratory infection | Acute infection/source diagnosis | Source-specific/surgical/other | Prospective/observational | Microbiology/clinical source diagnosis | Total/blood calprotectin | Plasma/blood | Gentian/PETIA/turbidimetric | 71 | 0.78 (0.67–0.86) | Exploratory/sensitivity: respiratory-site/pathogen-specific etiology comparison. |
| Hu et al., 2025 | China; Asia | Exploratory diagnostic AUC; Secondary mortality/survival AUC; Secondary mortality/survival 2×2 | Septic shock diagnosis | Sepsis diagnosis | Sepsis/critical illness prognosis | Observational/prognostic | 28-day mortality | Calprotectin/S100A8/A9-related | Blood/plasma/serum (as reported) | As reported | 335 | Exploratory severity/septic shock AUC: 0.74 (0.68–0.79); prognostic mortality/survival AUC: 0.78 (0.73–0.83), reported ROC AUC; threshold-based mortality/survival analysis used 2×2 data | Exploratory/sensitivity for diagnosis because the endpoint was S100A9/septic shock severity; contributes to reported-AUC mortality/survival synthesis and mortality/survival 2×2 analysis. |
| Huang et al., 2016 | China; Asia | Exploratory diagnostic AUC | Sepsis subgroup vs non-sepsis subgroup | Sepsis diagnosis | Postoperative surgical ICU | Prospective observational study | ACCP/SCCM 2003 sepsis criteria; new-onset sepsis within 7 postoperative days | Serum calprotectin | Serum | CALPROLAB ELISA ALP | 163 for sepsis vs non-sepsis endpoint; 213 total including healthy controls | 0.86 (0.67–0.98) | Exploratory/sensitivity: postoperative new-onset sepsis subgroup, not general ED/ICU suspected infection. |
| Jonsson et al., 2017 | Sweden; Europe | Main diagnostic AUC; Main diagnostic 2×2 | 24 h before confirmed infection vs no infection | Acute infection/source diagnosis | ICU/critical care | Prospective/observational | Expert adjudication/clinical probability | Total/blood calprotectin | Plasma/blood | Gentian/PETIA/turbidimetric | 110 | 0.82 (0.70–0.94) | Eligible for main analysis under clinical suspected infection/sepsis comparator principle. |
| Larsson et al., 2020 | Sweden; Europe | Main diagnostic AUC; Main diagnostic 2×2; Secondary mortality/survival AUC; Secondary mortality/survival 2×2 | Sepsis vs all non-sepsis ICU patients | Sepsis diagnosis | ICU/critical care | Prospective/observational | Sepsis criteria | Total/blood calprotectin | Plasma/blood | Gentian/PETIA/turbidimetric | 271 | Diagnostic AUC: 0.67 (0.60–0.74); prognostic mortality/survival AUC: 0.64 (0.56–0.72), reported ROC AUC; threshold-based mortality/survival analysis used 2×2 data | Eligible for main diagnostic analysis; also contributes to reported-AUC mortality/survival synthesis and mortality/survival 2×2 analysis. |
| Lengquist et al., 2025 | Sweden; Europe | Main diagnostic AUC; Main diagnostic 2×2 | Sepsis vs non-sepsis ICU patients | Sepsis diagnosis | ICU/critical care | Retrospective/biobank | Sepsis criteria | Total/blood calprotectin | Plasma/blood | Gentian/PETIA/turbidimetric | 4732 | 0.61 (0.59–0.62) | Eligible for main analysis under clinical suspected infection/sepsis comparator principle. |
| Liu et al., 2021 | China; Asia | Exploratory diagnostic AUC | Community-acquired pneumonia vs non-CAP/control subjects | Acute infection/source diagnosis | Respiratory and Critical Care Medicine inpatient CAP cohort plus physical-examination controls | Prospective cohort study | CAP diagnostic criteria; control subjects from physical examination center; severity by CAP scores | S100A9 | Serum | ELISA; human S100A9 kits from Wuhan Colorful Gene Biological Technology | 330 for CAP vs control endpoint; 220 CAP patients for severity/prognosis analyses | 0.79 (0.70–0.88) | Exploratory/sensitivity: CAP compared with healthy physical-examination controls; not a clinical suspected-infection comparator. |
| Sejersen et al., 2025b | Sweden; Europe | Overlapping cohort report; unique S100A8/S100A9 subunit results retained | Sepsis diagnosis | Sepsis diagnosis | ICU/critical care | Prospective observational study | Sepsis-3: suspected infection and SOFA >2 on ICU admission; 30-day mortality for prognosis | Total calprotectin; S100A8 and S100A9 subunits | EDTA plasma | Gentian PETIA/turbidimetric assay on Mindray BS380 for total calprotectin; R&D Systems ELISA for S100A8/S100A9 | 271 | Total-calprotectin diagnostic AUC 0.67 (0.60–0.74) and prognostic AUC 0.64 (0.56–0.72) were derived from the same samples and data as Larsson et al., 2020 and were not entered as independent estimates; unique S100A8/S100A9 subunit results were retained. | Same 271-patient ICU cohort as Larsson et al., 2020 for total-calprotectin endpoints; retained as a separate report only for unique S100A8/S100A9 analyte results. |
| Simm et al., 2016 | Germany; Europe | Exploratory diagnostic AUC; Exploratory diagnostic 2×2; Secondary mortality/survival AUC | Sepsis vs intoxication without inflammation | Sepsis diagnosis | ICU/critical care | Prospective/observational | Sepsis criteria | Total/blood calprotectin | Plasma/blood | Other/unclear assay | 38 | Exploratory diagnostic AUC: 0.95 (0.86–1.00); prognostic mortality/survival AUC: 0.70 (0.45–0.95), reported ROC AUC | Exploratory/sensitivity for diagnosis because of intoxication and postoperative special controls; contributes to reported-AUC mortality/survival synthesis. |
| Strambi et al., 2026 | Italy/Europe; Europe | Exploratory diagnostic AUC; Exploratory diagnostic 2×2 | IAI groups vs elective abdominal wall controls | Other | Source-specific/surgical/other | Prospective/observational | Microbiology/clinical source diagnosis | Total/blood calprotectin | Plasma/blood | Other/unclear assay | 151 | 0.90 (0.85–0.95) | Exploratory/sensitivity: intra-abdominal infection with elective surgical controls. |
| Venge et al., 2021 | Sweden; Europe | Exploratory diagnostic AUC | Mycoplasma pneumoniae vs viral respiratory infection | Acute infection/source diagnosis | Acute respiratory infection cohort; first visit before antibiotics (care setting not explicitly reported) | Observational diagnostic cohort (Bio-X cohort) | Confirmed acute respiratory infection etiology by clinical diagnosis plus microbiological/serological testing | Plasma calprotectin | EDTA plasma | Gentian calprotectin assay; analyzer/platform not specified | 61 for preferred mycoplasma vs viral calprotectin endpoint; 156 confirmed-infection patients overall | 0.87 (0.76–0.94) | Exploratory/sensitivity: pathogen-specific mycoplasma pneumoniae respiratory-infection comparison; not broad suspected infection/sepsis comparator. |
| Wollmer et al., 2021 | Sweden; Europe | Main diagnostic AUC | Final ED severe infection/pneumonia vs other dyspnea causes | Acute infection/source diagnosis | Emergency department/acute dyspnea | Observational ED cohort with prospective sampling | Primary ED diagnosis confirmed by research-nurse chart review with senior emergency physician support; acute infection/severe infection including pneumonia vs other dyspnea causes | Plasma calprotectin | EDTA plasma | Gentian calprotectin reagents on Mindray BS380 chemistry analyzer | 1287 | 0.78 (0.73–0.82) | Eligible for main analysis under clinical suspected infection/sepsis comparator principle. |
| Wollmer et al., 2026 | Sweden; Europe | Main diagnostic AUC | Sepsis/infection vs noninfectious SIRS | Sepsis diagnosis | Emergency department/acute care | Secondary analysis of prospective observational ED-Sepsis cohort | Infectious-disease physician chart review; outcomes were sepsis vs noninfectious SIRS and LRTI vs other infection sources | Plasma calprotectin | EDTA plasma | Gentian PETIA/turbidimetric assay on Mindray BS380 | 583 | 0.63 (0.55–0.71) | Eligible for main analysis under clinical suspected infection/sepsis comparator principle. |
| Zimmermann et al., 2026 | Switzerland/Europe; Europe | Main diagnostic AUC; Main diagnostic 2×2 | High-probability/confirmed infection vs no/low-probability infection | Acute infection/source diagnosis | ED/acute care | Prospective/observational | Expert adjudication/clinical probability | Total/blood calprotectin | Plasma/blood | Gentian/PETIA/turbidimetric | 245 | 0.53 (0.45–0.62) | Eligible for main analysis under clinical suspected infection/sepsis comparator principle. |

**Note:** Analysis role refers to the final evidence set used in the manuscript. Main diagnostic analyses were restricted to clinically relevant suspected-infection or suspected-sepsis comparator populations; studies with healthy controls, special postoperative or intoxication controls, pathogen- or site-specific designs, or severity-only endpoints were retained as exploratory, sensitivity, or secondary prognostic evidence as appropriate. Preferred AUC values follow the prespecified hierarchy: directly reported AUCs were prioritized, one clinically preferred endpoint was selected per study for each main synthesis, and overlapping cohort estimates were counted once per synthesis. Approximate analysed N reflects the endpoint-level sample and should not be summed as a unique participant total because analysis populations and reporting denominators varied. Abbreviations: AUC, area under the receiver operating characteristic curve; CAP, community-acquired pneumonia; CI, confidence interval; ECL, electrochemiluminescence; ED, emergency department; EDTA, ethylenediaminetetraacetic acid; ELISA, enzyme-linked immunosorbent assay; IAI, intra-abdominal infection; ICU, intensive care unit; LRTI, lower respiratory tract infection; NR, not reported; PCR, polymerase chain reaction; PETIA, particle-enhanced turbidimetric immunoassay; ROC, receiver operating characteristic; SIRS, systemic inflammatory response syndrome; SOFA, Sequential Organ Failure Assessment; UTI, urinary tract infection.

**Supplementary Table 2. Risk-of-bias and applicability assessment.**

| **Study** | **Quality assessment role** | **Supplementary prognostic ROB note** | **Patient selection - risk of bias** | **Index test - risk of bias** | **Reference standard - risk of bias** | **Flow and timing - risk of bias** | **Patient selection - applicability** | **Index test - applicability** | **Reference standard - applicability** | **Overall QUADAS-2 judgement** | **Rationale** |
| --- | --- | --- | --- | --- | --- | --- | --- | --- | --- | --- | --- |
| Bakalar et al., 2026 | Diagnostic QUADAS-2 | NR | Low | Unclear | Low | Unclear | Unclear | Low | Unclear | Unclear | Clinical comparator compatible with main analysis. |
| Bartakova et al., 2019 | Diagnostic QUADAS-2 | NR | Low | Unclear | Low | Unclear | Low | Low | Low | Unclear | Clinical comparator compatible with main analysis. |
| Chen et al., 2025 | Prognostic ROB note | Outcome: 28-day mortality; Tool note: QUADAS-2 is not designed for prognostic prediction; this is a supplementary prognostic-risk note.; Participants: Unclear; Predictor measurement: Low; Outcome measurement: Low; Confounding/statistical model: High; Overall judgement: Unclear; Rationale: Threshold-based prognostic performance was extracted, but prognostic modelling, confounding control, and outcome timing varied across studies. | Not applicable to diagnostic QUADAS-2; prognostic endpoint only | Not applicable to diagnostic QUADAS-2; prognostic endpoint only | Not applicable to diagnostic QUADAS-2; prognostic endpoint only | Not applicable to diagnostic QUADAS-2; prognostic endpoint only | Not applicable to diagnostic QUADAS-2; prognostic endpoint only | Not applicable to diagnostic QUADAS-2; prognostic endpoint only | Not applicable to diagnostic QUADAS-2; prognostic endpoint only | Not applicable to diagnostic QUADAS-2; prognostic endpoint only | Not applicable to diagnostic QUADAS-2; prognostic endpoint only |
| Christensen et al., 2022 | Diagnostic QUADAS-2 | NR | Low | Unclear | Low | Low | Low | Low | Low | Unclear | Clinical comparator compatible with main analysis. |
| Diehl-Wiesenecker et al., 2024 | Diagnostic QUADAS-2 | NR | Low | Unclear | Low | Unclear | Low | Low | Low | Unclear | Clinical comparator compatible with main analysis. |
| Gao et al., 2015 | Diagnostic QUADAS-2 | Outcome: 28-day mortality; Tool note: QUADAS-2 is not designed for prognostic prediction; this is a supplementary prognostic-risk note.; Participants: Unclear; Predictor measurement: Low; Outcome measurement: Low; Confounding/statistical model: High; Overall judgement: High; Rationale: Threshold-based prognostic performance was extracted, but prognostic modelling, confounding control, and outcome timing varied across studies. | High | Low | Low | Low | High | Low | Low | High | Moved to exploratory/sensitivity analysis because population/comparator was less aligned with the main clinical suspected infection/sepsis question. |
| Garcia de Guadiana-Romualdo et al., 2024 | Prognostic ROB note | Outcome: 28-day mortality; Tool note: QUADAS-2 is not designed for prognostic prediction; this is a supplementary prognostic-risk note.; Participants: Unclear; Predictor measurement: Unclear; Outcome measurement: Low; Confounding/statistical model: High; Overall judgement: Unclear; Rationale: Threshold-based prognostic performance was extracted, but prognostic modelling, confounding control, and outcome timing varied across studies. | Not applicable to diagnostic QUADAS-2; prognostic endpoint only | Not applicable to diagnostic QUADAS-2; prognostic endpoint only | Not applicable to diagnostic QUADAS-2; prognostic endpoint only | Not applicable to diagnostic QUADAS-2; prognostic endpoint only | Not applicable to diagnostic QUADAS-2; prognostic endpoint only | Not applicable to diagnostic QUADAS-2; prognostic endpoint only | Not applicable to diagnostic QUADAS-2; prognostic endpoint only | Not applicable to diagnostic QUADAS-2; prognostic endpoint only | Not applicable to diagnostic QUADAS-2; prognostic endpoint only |
| Gigliotti et al., 2025 | Diagnostic QUADAS-2 | Outcome: 28-day mortality; Tool note: QUADAS-2 is not designed for prognostic prediction; this is a supplementary prognostic-risk note.; Participants: Unclear; Predictor measurement: Low; Outcome measurement: Low; Confounding/statistical model: High; Overall judgement: High; Rationale: Threshold-based prognostic performance was extracted, but prognostic modelling, confounding control, and outcome timing varied across studies. | High | Low | Low | Unclear | High | Low | Unclear | High | Moved to exploratory/sensitivity analysis because population/comparator was less aligned with the main clinical suspected infection/sepsis question. |
| Havelka et al., 2020 | Diagnostic QUADAS-2 | NR | High | Low | Low | Low | High | Low | Low | High | Moved to exploratory/sensitivity analysis because population/comparator was less aligned with the main clinical suspected infection/sepsis question. |
| Hu et al., 2025 | Diagnostic QUADAS-2 | Outcome: 28-day mortality; Tool note: QUADAS-2 is not designed for prognostic prediction; this is a supplementary prognostic-risk note.; Participants: Low; Predictor measurement: Low; Outcome measurement: Low; Confounding/statistical model: High; Overall judgement: Unclear; Rationale: Threshold-based prognostic performance was extracted, but prognostic modelling, confounding control, and outcome timing varied across studies. | High | Unclear | Low | Unclear | High | Low | Low | High | Moved to exploratory/sensitivity analysis because population/comparator was less aligned with the main clinical suspected infection/sepsis question. |
| Huang et al., 2016 | Diagnostic QUADAS-2 | NR | High | Unclear | Low | Unclear | High | Low | Low | High | Moved to exploratory/sensitivity analysis because population/comparator was less aligned with the main clinical suspected infection/sepsis question. |
| Jonsson et al., 2017 | Diagnostic QUADAS-2 | NR | Low | Low | Low | Low | Low | Low | Low | Low | Clinical comparator compatible with main analysis. |
| Larsson et al., 2020 | Diagnostic QUADAS-2 | Outcome: 30-day mortality; Tool note: QUADAS-2 is not designed for prognostic prediction; this is a supplementary prognostic-risk note.; Participants: Low; Predictor measurement: Low; Outcome measurement: Low; Confounding/statistical model: High; Overall judgement: Unclear; Rationale: Threshold-based prognostic performance was extracted, but prognostic modelling, confounding control, and outcome timing varied across studies. | Low | Low | Low | Low | Low | Low | Low | Low | Clinical comparator compatible with main analysis. |
| Lengquist et al., 2025 | Diagnostic QUADAS-2 | NR | Unclear | Low | Low | Unclear | Low | Low | Low | Unclear | Clinical comparator compatible with main analysis. |
| Liu et al., 2021 | Diagnostic QUADAS-2 | NR | High | Unclear | Low | Unclear | High | Low | Low | High | Moved to exploratory/sensitivity analysis because population/comparator was less aligned with the main clinical suspected infection/sepsis question. |
| Sejersen et al., 2025b | Diagnostic QUADAS-2 | NR | Low | Unclear | Low | Unclear | Low | Low | Low | Unclear | Clinical comparator compatible with main analysis. |
| Simm et al., 2016 | Diagnostic QUADAS-2 | NR | High | Unclear | Low | Low | High | Low | Low | High | Moved to exploratory/sensitivity analysis because population/comparator was less aligned with the main clinical suspected infection/sepsis question. |
| Strambi et al., 2026 | Diagnostic QUADAS-2 | NR | High | Unclear | Low | Low | High | Low | Low | High | Moved to exploratory/sensitivity analysis because population/comparator was less aligned with the main clinical suspected infection/sepsis question. |
| Venge et al., 2021 | Diagnostic QUADAS-2 | NR | High | Unclear | Low | Unclear | High | Low | Unclear | High | Moved to exploratory/sensitivity analysis because population/comparator was less aligned with the main clinical suspected infection/sepsis question. |
| Wollmer et al., 2021 | Diagnostic QUADAS-2 | NR | Low | Unclear | Low | Unclear | Low | Low | Low | Unclear | Clinical comparator compatible with main analysis. |
| Wollmer et al., 2026 | Diagnostic QUADAS-2 | NR | Low | Unclear | Low | Unclear | Low | Low | Low | Unclear | Clinical comparator compatible with main analysis. |
| Zimmermann et al., 2026 | Diagnostic QUADAS-2 | NR | Low | Low | Low | Low | Low | Low | Low | Low | Clinical comparator compatible with main analysis. |

**Note:** Diagnostic studies were assessed using QUADAS-2 across patient selection, index test, reference standard, and flow/timing domains. Prognostic-only studies were not assigned diagnostic QUADAS-2 judgments and were instead summarized with a supplementary prognostic risk-of-bias note. Applicability concerns were judged in relation to the main clinical suspected-infection/sepsis question. Abbreviations: NR, not reported; QUADAS-2, Quality Assessment of Diagnostic Accuracy Studies-2; ROB, risk of bias.

**Supplementary Table 3. GRADE certainty of evidence by evidence body.**

| **Outcome/evidence body** | **Studies** | **Effect estimate** | **Risk of bias** | **Inconsistency** | **Indirectness** | **Imprecision** | **Publication bias** | **Certainty** | **Interpretation** |
| --- | --- | --- | --- | --- | --- | --- | --- | --- | --- |
| Main diagnostic AUC, all eligible clinical diagnostic endpoints | 10 | 0.741 (0.657–0.810) | Serious | Very serious (I²=94.6%) | Serious: sepsis and acute infection/source diagnosis were clinically related but not identical target conditions | Not serious for pooled AUC, but confidence intervals varied across studies | Undetected/unclear; formal tests underpowered | Very low | Calprotectin/S100A8/A9 showed moderate overall discriminatory ability, but heterogeneity and target-condition diversity limit certainty. |
| Main diagnostic AUC: sepsis diagnosis | 4 | 0.703 (0.600–0.789) | Serious | Very serious (I²=89.3%) | Serious: sepsis definitions, settings, and comparator groups varied | Serious: only four independent cohorts | Unclear | Very low | The sepsis-specific AUC was modest and should not be interpreted as sufficient for stand-alone diagnosis. |
| Main diagnostic AUC: acute infection/source diagnosis | 6 | 0.758 (0.636–0.849) | Serious | Very serious (I²=92.6%) | Serious: ED, ICU, burn, and pathogen/source contrasts were not interchangeable | Serious | Unclear | Very low | Discrimination appeared higher than in sepsis diagnosis, but this may reflect spectrum and reference-standard differences. |
| Main 2×2: sepsis diagnosis | 2 | Sensitivity 0.70 (0.46–0.86); specificity 0.57 (0.55–0.59) | Serious | Very serious for sensitivity (I²=92.2%) | Serious | Very serious: only two studies | Not assessable | Very low | Threshold-specific sensitivity and specificity are highly uncertain. |
| Main 2×2: acute infection/source diagnosis | 3 | Sensitivity 0.46 (0.09–0.88); specificity 0.86 (0.60–0.96) | Serious | Very serious (sensitivity I²=98.7%; specificity I²=91.0%) | Serious | Very serious: only three studies and wide intervals | Not assessable | Very low | Specificity was relatively high, but sensitivity was unstable and threshold dependent. |
| Exploratory broader diagnostic AUC | 19 | 0.794 (0.735–0.843) | Very serious | Very serious (I²=93.9%) | Very serious: includes special controls, pathogen/site-specific studies, and exploratory endpoints | Serious | Unclear | Very low | Higher exploratory AUC should be interpreted as hypothesis-generating rather than confirmatory. |
| Secondary mortality/survival threshold performance (2×2 data) | 6 | Sensitivity 0.72 (0.65–0.77); specificity 0.66 (0.53–0.77) | Serious/very serious | Serious for specificity (I²=93.7%) | Serious: prognostic outcome, different thresholds and analytes | Serious | Unclear | Very low | Threshold-based 2×2 data suggest possible prognostic signal, but cut-offs, outcome horizons, analytes, and specificity were heterogeneous. |
| Secondary mortality/survival AUC (reported ROC AUCs) | 8 | AUC 0.737 (0.688–0.786) | Serious/very serious: several studies were prognostic cohorts or exploratory diagnostic cohorts with variable adjustment and patient spectrum | Serious (I² = 63.6%) | Serious: mortality/survival endpoints varied by population, analyte, sampling time, and outcome horizon | Serious: eight independent cohorts, with varying confidence intervals and endpoint definitions | Unclear; formal tests are underpowered and prognostic AUC reporting may be selective | Very low | Reported AUCs suggest moderate prognostic discrimination, but the evidence is heterogeneous and insufficient for stand-alone prognostic use. |

Note: GRADE certainty was assessed by evidence body rather than by individual study. The mortality/survival threshold-performance row corresponds to Figure 6 and Supplementary Figure S10 and is based on 2×2 sensitivity/specificity data. The mortality/survival AUC row is based on eight independent cohorts with directly reported ROC AUCs, not AUCs derived from the 2×2 threshold table; the corresponding graphical displays are Supplementary Figure S11. Abbreviations: AUC, area under the receiver operating characteristic curve; CI, confidence interval; ED, emergency department; GRADE, Grading of Recommendations Assessment, Development and Evaluation; ICU, intensive care unit; I², inconsistency statistic; ROC, receiver operating characteristic.
