# Supplementary material for: Circulating calprotectin and S100A8/A9-related biomarkers for diagnosis and prognostic stratification in adult acute infection and sepsis: a systematic review and meta-analysis
Source: Front Cell Infect Microbiol. 2026 Oct 14;16:1934020. doi: 10.3389/fcimb.2026.1934020 (PMC13713635; doi:10.3389/fcimb.2026.1934020)
Supplement: Supplementary file 4 [file Supplementaryfile4.docx]

# **Supplementary File 4. Supplementary Methods**

# **Eligibility and data selection**

## **Detailed eligibility criteria**

Eligibility was defined according to the Population, Index test, Comparator, Outcomes, and Study design framework. Eligible populations comprised adults aged 18 years or older assessed in emergency departments, intensive care units, hospital wards, perioperative acute-care settings, or other relevant acute-care environments for suspected or confirmed acute infection, bacterial infection, sepsis, septic shock, or clinically plausible non-infectious inflammatory mimics.

Eligible index tests were blood, plasma, or serum total calprotectin, the S100A8/A9 heterodimer, S100A8, or S100A9. Baseline or early presentation/admission measurements were prioritized for diagnostic analyses. Serial measurements, clearance, and delta values were considered separately for prognostic analyses when reported.

Diagnostic outcomes included acute infection, bacterial infection, sepsis, septic shock, culture-confirmed bloodstream infection, lower respiratory tract infection, and infection-source differentiation. Prognostic outcomes included mortality/survival and severity-related endpoints. Outcomes were defined using clinical adjudication, microbiology, consensus sepsis criteria, or composite reference standards. Eligible designs included prospective or retrospective diagnostic accuracy studies, cohort studies, nested case-control studies, clinical prediction studies, and prognostic studies reporting original patient-level or study-level data.

Pediatric-only, animal, in-vitro, fecal or local-fluid calprotectin studies without circulating measurements, narrative reviews, editorials without original data, and studies without extractable diagnostic or prognostic performance data were excluded. Mixed-age studies were eligible only when adult data were separable or adults constituted the overwhelming majority of the study population.

## **Definition of the main and exploratory evidence sets**

The main diagnostic evidence set was restricted to clinically relevant suspected-infection, suspected-sepsis, non-infectious inflammatory, viral, non-bacterial, or low-probability infection comparator populations. Healthy-only controls were not considered clinically representative comparators for the main diagnostic question.

Studies using healthy volunteers, elective surgical controls, intoxication controls, single-pathogen comparisons, highly site-specific case-control designs, or severity- or shock-related endpoints were retained in the systematic review but assigned to broader exploratory or sensitivity analyses when they did not meet the main clinical-comparator criteria. Each study contributed only once to a given primary synthesis. A study could contribute to different diagnostic, prognostic, or exploratory evidence sets when it reported distinct eligible outcomes, but the same endpoint was not entered more than once into the same synthesis. When multiple reports used the same cohort and biomarker endpoint, the estimate was entered only once in a given synthesis; secondary reports were retained only for unique analytes or outcomes.

## **Detailed data extraction**

Two reviewers (YL and YD) independently extracted data using a standardized form. Extracted variables included country or region, clinical setting, study design, population and comparator characteristics, reference standard, target condition, analyte, specimen type, sampling time, assay method, sample size, diagnostic or prognostic endpoint, reported receiver operating characteristic AUC with its 95% confidence interval (CI), cut-off value, true-positive, false-positive, false-negative, and true-negative counts, sensitivity, specificity, and prognostic outcome data. Disagreements were resolved by consensus or, when necessary, by consultation with a third reviewer (GC).

AUCs were included only when reported in the article or supplementary material. When an AUC was reported without a CI or standard error, its standard error and 95% CI were estimated using the Hanley–McNeil approximation based on the reported AUC and the numbers of participants with and without the target condition. Threshold-based 2×2 counts were extracted directly or reconstructed from reported group sizes and sensitivity and specificity when the reconstructed counts were internally consistent after allowing for rounding.

## **Endpoint and threshold selection**

To avoid double counting, one clinically prioritized endpoint was selected from each study for each primary diagnostic or prognostic synthesis. When multiple diagnostic AUCs were available, the endpoint most closely aligned with the prespecified clinical question was selected for the main analysis; other eligible endpoints were retained for exploratory or subgroup analyses.

For threshold-based analyses, one clinically relevant author-reported threshold was selected per study and outcome, and alternative thresholds were not entered simultaneously into the same synthesis. Mortality/survival outcomes were kept separate from diagnostic outcomes. Septic shock and other severity-related endpoints that did not address the main diagnostic question were retained only for exploratory or descriptive analyses.

For comparisons with other biomarkers, only external single-marker AUCs reported for the same clinical endpoint were extracted, and combined models were excluded from single-marker pooling. Combined-model analyses included models explicitly adding calprotectin or S100A8/A9 to clinical assessment, C-reactive protein, another biomarker, or an existing clinical model. Prognostic combined-model results were not pooled because only one eligible model was extractable.

# **Statistical analysis**

## **AUC meta-analysis**

All statistical analyses and figure generation were performed using R version 4.6.0 (R Foundation for Statistical Computing, Vienna, Austria). Diagnostic AUCs and directly reported prognostic AUCs were synthesized separately using random-effects models after logit transformation and were back-transformed for presentation with 95% CIs. Heterogeneity was summarized using I² and interpreted cautiously when few studies contributed.

The main diagnostic AUC evidence set was first synthesized as an overall descriptive analysis of eligible clinical diagnostic endpoints and was then stratified by target condition: sepsis diagnosis versus acute infection or infection-source-related diagnosis. Because the target-condition comparison was exploratory, numerical differences between strata were not interpreted as formal between-subgroup interactions. Directly reported prognostic AUCs were synthesized separately from threshold-based prognostic sensitivity and specificity analyses.

## **Threshold-based diagnostic analyses**

For studies with extractable 2×2 data, sensitivity and specificity were jointly synthesized using bivariate diagnostic random-effects models based on the Reitsma approach. Summary receiver operating characteristic (SROC) plots displayed study-level operating points and summary estimates where data permitted.

Likelihood-ratio scattergrams illustrated potential rule-in and rule-out performance. Fagan-style plots translated pooled likelihood ratios into illustrative post-test probabilities at pretest probabilities of 10%, 30%, and 50%; these probabilities were used for illustration rather than as fixed clinical prevalence estimates. Threshold-effect plots explored whether variation in reported cut-offs might contribute to the sensitivity–specificity trade-off. Galbraith plots were used as exploratory displays of studies contributing disproportionately to diagnostic-odds-ratio heterogeneity. Deeks’ funnel plot asymmetry test explored small-study effects or publication bias when estimable (Deeks et al., 2005). These analyses were interpreted cautiously because several evidence sets contained few studies.

## **Exploratory subgroup and stability analyses**

Exploratory AUC subgroup analyses were conducted when relevant study-level covariates were available. The examined variables included target condition, availability of extractable 2×2 data, clinical setting, and reference standard.

Available main-analysis 2×2 studies were also considered together in a descriptive pool and explored according to cut-off category, region, clinical setting, target condition, and reference standard. Because this pool included different diagnostic targets and thresholds, it was not treated as a clinically interchangeable population. Subgroup estimates were not interpreted as formal tests of interaction; single-study strata were displayed descriptively but were not interpreted as pooled estimates.

Exploratory stability analyses examined whether the broader diagnostic estimate was driven by specific study designs or individual studies. Starting from the main clinical-comparator evidence set, the following categories were added sequentially, with each study contributing once: special-comparator or postoperative designs; pathogen- or respiratory-specific designs; site- or bacteremia-specific designs; and severity- or shock-related endpoints. Leave-one-group-out analyses sequentially removed each design category from the broader evidence set, and leave-one-study-out analyses sequentially excluded individual studies. These analyses assessed directional stability and were not interpreted as formal interaction tests.

## **Single-biomarker comparator analyses**

External single-biomarker AUCs were eligible only when the comparator and calprotectin/S100A8/A9 were evaluated for the same clinical endpoint within the same study. Diagnostic and prognostic comparator AUCs were synthesized separately using random-effects models on the logit AUC scale.

For head-to-head comparisons, the within-study AUC difference was calculated as the comparator AUC minus the calprotectin/S100A8/A9 AUC and was pooled when at least two studies reported the same comparison. Most reports did not provide the covariance required for formal paired DeLong comparisons. Standard errors of AUC differences were therefore approximated conservatively from the two reported AUC standard errors, and the resulting confidence intervals and P values were interpreted as supportive rather than definitive tests of biomarker superiority.

Illustrative summary ROC curves were reconstructed from pooled AUCs using an equal-variance binormal approximation. These curves visualized relative discrimination and were not patient-level ROC curves, SROC curves derived from sensitivity and specificity data, or additional meta-analytic estimates.

## **Combined-model analyses**

Combined-model analyses were descriptive because model composition and clinical endpoints varied across studies and several combined-model AUCs lacked CIs. For visualization, ROC curves were reconstructed from the mean AUCs of calprotectin alone, the base marker or model, and the base-plus-calprotectin model using an equal-variance binormal approximation. These reconstructed curves were relative displays and were not interpreted as patient-level SROC curves.

For each extractable endpoint, incremental change was calculated as the AUC of the base-plus-calprotectin model minus the AUC of the corresponding base marker or model. No formal pooled incremental-effect estimate was calculated.

## **Prognostic analyses**

Prognostic analyses were conducted separately from diagnostic analyses. Directly reported prognostic AUCs were synthesized independently from threshold-based prognostic sensitivity and specificity; a reported prognostic AUC was not replaced by an AUC approximated from threshold-based 2×2 data.

Threshold-based mortality/survival sensitivity and specificity were jointly summarized using a separate bivariate 2×2 framework. Supporting SROC, Fagan-style, threshold-effect, and Deeks plots were generated using the same general procedures as those applied to diagnostic analyses.

All prognostic analyses were considered exploratory because studies differed in analyte, specimen type, cut-off value, sampling time, outcome horizon, patient population, and adjustment for potential confounding. Prognostic combined models were not pooled because only one eligible model was extractable.
